# Endoscopic decompression of acute intestinal distension is associated with reduced mortality in critically ill patients

**DOI:** 10.1186/s12876-020-01233-y

**Published:** 2020-04-06

**Authors:** Thorsten Book, Martha M. Kirstein, Andrea Schneider, Michael P. Manns, Torsten Voigtländer

**Affiliations:** Department of Gastroenterology, Hepatology and Endocrinology, Hannover Medical School, Carl-Neuberg Str. 1, 30625 Hannover, Germany

**Keywords:** Endoscopic decompression, Ileus, Transanal tube, Pseudo-obstruction, Critically ill patients, Impaired intestinal transit, Ogilvie syndrome

## Abstract

**Background:**

Endoscopic placement of intestinal decompression tubes is a feasible technique for treatment of acute intestinal dilation. Given the heterogeneity of the underlying diseases leading to intestinal obstruction data on the significance of endoscopic procedures for treatment of these conditions are sparse.

**Methods:**

In the study period from 2008 to 2019 all patients receiving a decompression tube were identified by retrospective chart review and analyzed.

**Results:**

A total of 59 decompression tubes were placed in 50 patients. Technical success was achieved in 98% (58/59 tubes). As major complication one small bowel perforation occurred (1/59; 1.7%). Causes for impaired intestinal transit comprised tumor stenoses 22% (11/50), infections 18% (9/50), post-operative paralysis 14% (7/50), neurological diseases 8% (4/50), trauma 2% (1/50) and others 36% (18/50). Most patients (74%; 37/50) were critically ill and treated on intensive care unit. Treatment response after tube insertion was documented in 76% of patients (38/50) whereas 24% (12/50) did not fulfill response criteria. Patients with treatment response showed a significantly better outcome compared to non-responders. Responders had a median survival of 113 days (95% CI 41–186) compared to 15 days (95% CI 6–24) in non-responders (*p* = 0.002). Analysis of laboratory parameters after stratification in responders and non-responders to endoscopic therapy showed that non-responders had significantly higher levels of CRP and lower platelet count at baseline (CRP 262 mg/L (IQR 101–307) vs. 94 mg/L (IQR 26–153): *p* = 0.027; platelets 69 thsd/μL (IQR 33–161) vs. 199 thsd/μL (IQR 138–289): *p* = 0.009).

**Conclusions:**

Endoscopic decompression is a safe procedure for acute management of impaired intestinal transit even in critically ill patients. Response to therapy is associated with improved outcome and markers of inflammation and organ function such as CRP, platelet count and serum lactate have to be taken into account for therapy monitoring and evaluation of prognosis.

## Background

Endoscopic placement of intestinal decompression tubes is a feasible technique for treatment of acute intestinal dilation [1–3]. A variety of diseases including malignant, benign and pseudo-obstructive conditions may lead to impaired intestinal transit reflected by dilated intestine in abdominal imaging studies [3, 4]. Mechanistically, increasing wall distension of the intestine leads to augmented transmural pressure promoting disturbance of microcirculation with local hypoxia, interstitial edema and bacterial translocation [4]. Endoscopic decompression reduces intestinal wall tension, intraabdominal pressure and facilitates intermittent drainage [4, 5]. Given the heterogeneity of the underlying diseases leading to intestinal obstruction or pseudo-obstruction data on the significance of endoscopic procedures for treatment of these conditions are sparse [5]. This prompted us to analyze patients receiving an endoscopic decompression tube regarding technical and clinical success of the intervention and long-term outcome. Special emphasis was placed on identification of prognostic factors for treatment success.

## Methods

In the study period from January 2008–December 2019 patients receiving a decompression tube were identified by retrospective chart review. The diagnosis of impaired intestinal transit was verified by review of our clinical database and imaging studies. Demographics, laboratory and medical data including outcome parameters were retrieved from the clinical and endoscopic data base. Patients with clinical and/or radiological response were compared to patients without treatment response. Treatment response was assessed 7–10 days after endoscopic tube placement by chart review and analysis of the available clinical documentation (routine patient care). Radiological response was defined as documented reduction in intestinal diameter in imaging studies during follow-up (at least 25% reduction in intestinal diameter). Clinical response was defined as improved general condition with de-escalation of medical therapy (analgetics and/or prokinetic agents) and start of enteral nutrition. Successful endoscopic decompression as bridging therapy to a scheduled operation was rated as treatment response. All endoscopic procedures were performed by an experienced endoscopist. Radiological findings were analyzed by two radiologists. The study protocol was approved by the local institutional ethics review board and is in accordance with the Declaration of Helsinki. Written informed consent was obtained from all patients or their legal representatives.

### Tube placement

For endoscopic decompression of the colon, a routine colonoscope or alternatively a paediatric colonoscope was used. In the upper gastrointestinal tract, endoscopy was performed with a routine gastroscope. Generally, the procedure was performed under fluoroscopy, only in a few exceptions the endoscopy had to be performed without fluoroscopy. Patients were sedated on intensive care unit with different regimens depending on the clinical situation. In the endoscopy unit, sedation was performed at the discretion of the anaesthesiologist (gas or intravenous anaesthesia). Under cautious and restrictive CO2 insufflation, the endoscope was advanced to the distended intestinal parts under fluoroscopic control. Stool residues were mobilized with an endowasher. A standard guide wire was placed in the dialted intestine. In case of a severe stenosis the guide wire was advanced over the stricture and the anatomical position was checked under fluoroscopy/application of contrast agent. After removal of the endoscope, the wire remained and served as a guide for the decompression tube. For decompression, a flexible 3-lm silicone drainage was used (16 French/length 2400 mm or 22 French/length 2000 mm (transanal tube); Create medic Co., Ltd., Yokohama, Japan). The decompression tube was carefully inserted into the distended intestinal section via the guide wire under repeated fluoroscopic control. After blocking of the balloon a final fluoroscopic position check was performed and an intermittent suction was connected to achieve decompression.

Statistical analyses were performed using SPSS 24.0 (SPSS Inc., Chicago, IL, USA). Data were expressed as number/percentages or median with interquartile range (IQR). Differences between categorical variables were calculated using Pearson’s Chi-squared test. OS was assessed using the Kaplan-Meier estimation. Comparison was made using the Log rank (Mantel-Cox) test. A probability (p) value less than 0.05 was considered significant.

## Results

**Fig. 1:**
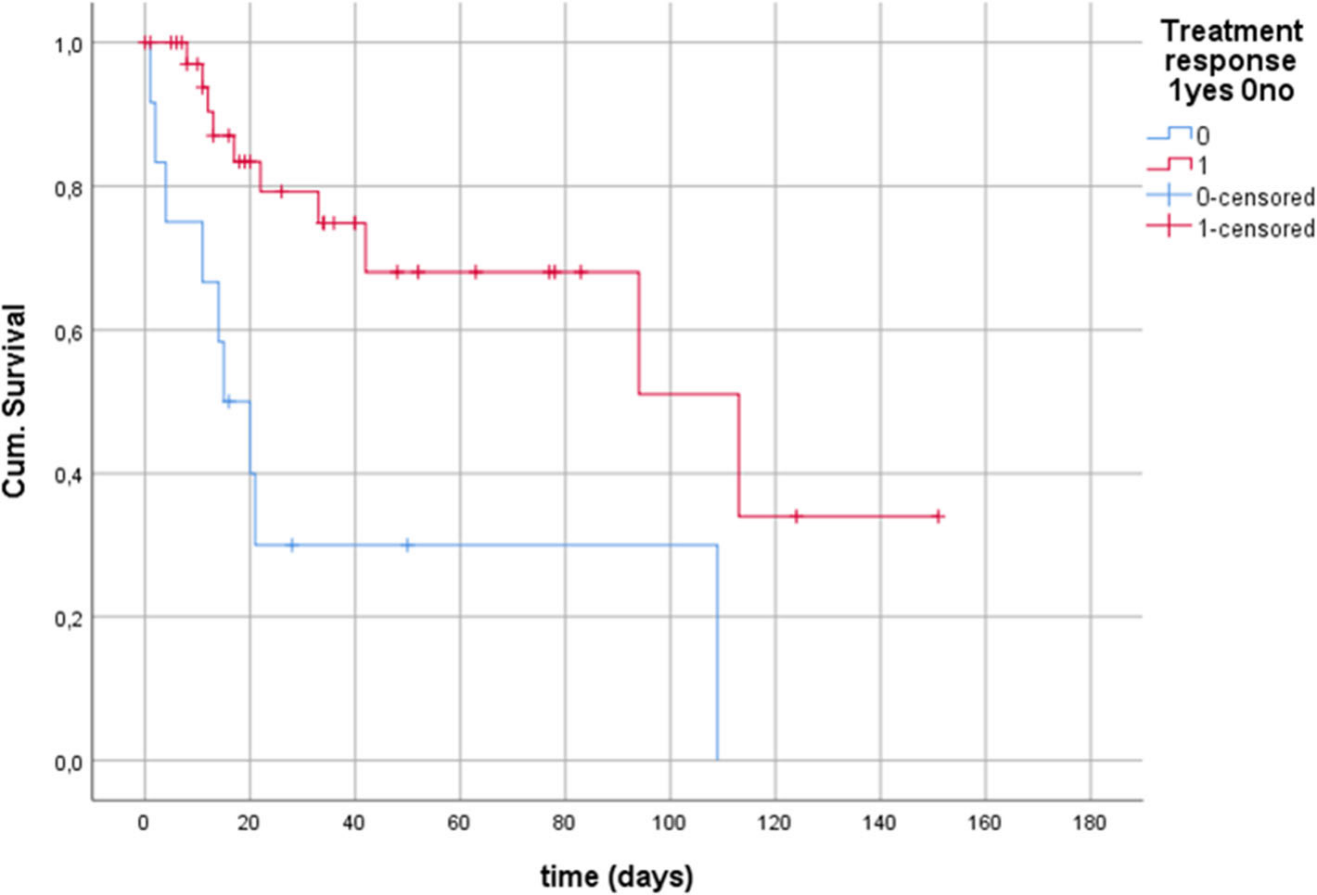
Survival of patients with treatment response and non-response to endoscopic decompression therapy. Patients with treatment response showed a significantly better outcome compared to non-responders. Responders had a median survival of 113 days (95% CI 41–186) compared to 15 days (95% CI 6–24) in non-responders (p = 0.002) (Fig. 1)

A total of 59 decompression tubes were placed in 50 patients during the study period. Technical success was achieved in 98% (58/59 tubes) of interventions. As major complication one small bowel perforation occurred (1/59; 1.7%). The perforation was facilitated by inflamed tissue. Three tubes dislocated during follow-up and were replaced (3/59; 5.1%). No other severe complication was documented. 61% (36/59) of tubes were placed in the colon and 39% (23/59) in the upper gastrointestinal tract (21/23; 91.3% jejunum, 2/23; 8.7% duodenum). In 6 patients a tube was inserted in the upper and lower gastrointestinal tract concomitantly. Seven of the 36 tubes of the colon were placed due to malignant obstruction (19.4%). Other indications comprised impaired transit due to infections (7/36; 19.4%), post-surgery (16.7%; 6/36), neurological diseases (11.1%; 4/36) and miscellaneous diseases (33.3%; 12/36). Median time from diagnosis of significant bowel dilatation to tube placement was 1 day (IQR 0–6). Median follow-up was 20 days (IQR 11–42). Prokinetic medication was administered in 40% of patients (20/50). The majority of patients was male (62%; 31/50) with a median age of 54 years (IQR 39–68). Demographic and laboratory parameters are given in Table 1. A paralytic ileus was detected in 56% of patients (28/50) whereas 34% (17/50) of patients had a mechanical bowel obstruction (10%, 5/50 other reasons). Causes for impaired intestinal transit comprised tumor stenoses in 22% (11/50), infections 18% (9/50), post-operative paralysis 14% (7/50), neurological diseases 8% (4/50), trauma 2% (1/50) and others 36% (18/50). Most patients (74%; 37/50) were critically ill and treated on intensive care unit (ICU). Median stay on ICU was 14 days (IQR 0–37). Half of the patients received vasopressor therapy (50%; 25/50) and 62% of patients (31/50) were mechanically ventilated. Treatment response after tube insertion was documented in 76% of patients (38/50) whereas 24% (12/50) did not fulfill the response criteria. Patients with treatment response showed a significantly better outcome compared to non-responders. Responders had a median survival of 113 days (95% CI 41–186) compared to 15 days (95% CI 6–24) in non-responders (*p* = 0.002) (Fig. 1). Etiology of intestinal transit failure (pseudo-obstruction versus obstruction) did not affect the outcome of patients who underwent endoscopic decompression (*p* = 0.741). In the cohort 24% (12/50) underwent endoscopic decompression as bridging to surgery. Analysis of laboratory parameters after stratification in responders and non-responders to endoscopic therapy showed that non-responders had significantly higher levels of CRP and lower platelet count at baseline (CRP 262 mg/L (IQR 101–307) vs. 94 mg/L (IQR 26–153): *p* = 0.027; platelets 69 thsd/μL (IQR 33–161) vs. 199 thsd/μL (IQR 138–289): *p* = 0.009). After 14 days non-responders had significantly higher levels of lactate (2.8 mmol/L (IQR 1.7–11.2) vs. 0.9 mmol/L (IQR 0.7–1.2): *p* = 0.004) (Table 2).

**Table 1:** Demographic, clinical and laboratory parameters of patients who received an endoscopic decompression tube

	Median/number	IQR (25–75)
Age (years)	54	39–68
Gender		
Female	19	
Male	31	
Body mass index (kg/m^2^)	24.5	20.4–29.2
Follow up (days)	20	11–42
Placement of tube after first diagnosis (days)	1	0–6
Length of hospital stay (days)	37	19–68
Treatment on ICU (days)	14	0–37
Mechanical ventilation (days)	4	0–25
Renal replacement therapy		
No	41	
Yes	8	
Opiods		
No	30	
Yes	19	
Cause of intestinal obstruction		
Trauma	1	
Infection	9	
Neurological	4	
Operation	7	
Tumor	11	
Others	18	
Laboratory parameters at first diagnosis		
Leukocytes (n/μL)	6715	4800–12,900
Platelets (Thsd/μL)	182	88–271
Hemoglobin (g/dL)	9,9	8.6–11.5
INR (Ratio)	1.15	1.04–1.34
CRP (mg/L)	107	29–248
PCT (μg/L)	1	0–3.3
Creatinine (μmol/L)	91	61–169
Lactate (mmol/L)	1,1	0.8–2.2
Bilirubin (μmol/L)	17	19,906
Lactate dehydrogenase (U/L)	262	172–394
Alkaline phospahatse (U/L)	95	78–256
Gamma-glutamyl transferase (U/L)	63	34–401
Alanine aminotransferase (U/L)	24	13–65

**Table 2:** Laboratory parameters of non-responders (left panel) and responders (right panel) to endoscopic decompression therapy at baseline, after 3–7 days and 14 days

	No treatment response		Treatment response		
	Median	IQR (25–75)	Median	IQR (25–75)	***p***-value
Leukocytes (n/μL)	4050	1600–8250	7500	5800–14,600	**0.018**
Platelets (Thsd/μL)	69	33–161	199	138–289	**0.009**
CRP (mg/L)	262	101–307	94	26–153	**0.027**
Bilirubin (μmol/L)	28	8–100	17	7–45	0.456
Lactate (mmol/L)	1.2	0.9–2.2	1.1	0.7–1.9	0.391
Leukocytes (n/μL) 3–7 days	3400	2700–9200	9000	5900–11,400	0.054
Platelets (Thsd/μL) 3–7 days	106	17–204	172	114–282	0.054
CRP (mg/L) 3–7 days	202	38–324	60	27–167	0.141
Leukocytes (n/μL) 14 days	5800	1900–11,800	8700	6500–9800	0.37
Platelets (Thsd/μL) 14 days	200	65–217	294	167–369	0.346
CRP (mg/L) 14 days	120	58–283	61	38–125	0.116
Lactate (mmol/L) 14 days	2.8	1.7–11.2	0.9	0.7–1.2	**0.004**

## Discussion

Ileus is an occlusion or paralysis of the bowel preventing forward passage of intestinal contents leading to potentially life-threatening complications [6]. Surgery in the setting of acute ileus is associated with a complication rate of up to 75% and high mortality rates of 40% [3, 7, 8]. Minimal-invasive endoscopic techniques may lead to an acute decompression of intestinal dilation facilitating a scheduled operation or conservative management of patients. Our study was not designed to analyze the concept of performing surgery after acute endoscopic decompression of the intestine. No standardized algorithm or pre-defined clinical endpoint was used for the selection of patients who receive a decompression tube. This decision was based on the physicians’ discretion. Nevertheless, our data confirm that endoscopic decompression is often successful even in critically ill patients showing low complication rates. The majority of patients was treated on ICU with mechanical ventilation and vasopressor therapy. Even in this highly selected patient cohort only one perforation occurred. Most importantly we show a high treatment response to endoscopic therapy. In 76% of patients treatment response was documented and response was also associated with improved outcome. Comparably, Fischer et al. showed a treatment response or conversion of an emergency clinical situation to semielective treatment in 73% of patients [3]. However, Fischer et al. described a more homogenous patient cohort of patients with malignant stenoses of the colon [3]. Our cohort is characterized by a mixture of benign and malignant causes for impaired intestinal transit. Nevertheless, we demonstrate similar results underlining that endoscopic tube placement for decompression is effective and safe in various entities of impaired intestinal transit. Consequently, it should be considered in early stages of ileus for decompression and de-escalation of emergency clinical situations. In our cohort, non-responders to endoscopic therapy had higher levels of CRP and lower platelet counts at baseline. Both findings seem reasonable as CRP is a surrogate parameter of inflammation and is widely used to detect severe infections [9]. The predictive value of CRP varies among studies, however its potential use in detection of inflammation and/or infection is generally accepted [9, 10]. An elevation of CRP reflects an activation of immune responses which are often triggered by infections in critically ill patients leading to worse outcome [11]. Thrombocytopenia is the most common hemostatic disorder in patients admitted to ICUs [12]. Mechanisms contributing to a decrease in platelet count in critically ill patients are multifactorial, among which sepsis and trauma are the most frequent. A low platelet count is a strong independent predictor of morbidity and mortality as it is associated with life-threatening bleeding or thrombosis [12]. Non-responders to endoscopic therapy also showed elevated serum lactate levels 14 days after tube placement. Serum lactate is an independent predictor of in-hospital mortality in ICU patients [13]. Thus the elevated serum lactate reflects the disease severity and insufficient treatment reponse in this subgroup of patients receiving decompression tubes.

## Conclusions

In summary, we show that endoscopic decompression is a feasible and safe procedure for acute management of impaired intestinal transit even in criticall ill patients. Response to therapy is associated with improved outcome and markers of inflammation and organ function such as CRP, platelet count and serum lactate have to be taken into account for therapy monitoring and evaluation of prognosis. Prospective studies are needed to confirm these results and to minimize confounding factors.

## Data Availability

Data can be provided by the corresponding author upon request and will be uploaded to a data repository.

## Abbreviations

IQR: Interquartile range
*p* value: probability value
ICU: intensive care unit
